# Haitian and international responders' and decision-makers' perspectives regarding disability and the response to the 2010 Haiti earthquake

**DOI:** 10.3402/gha.v8.27969

**Published:** 2015-08-07

**Authors:** Matthew R. Hunt, Ryoa Chung, Evelyne Durocher, Jean Hugues Henrys

**Affiliations:** 1 School of Physical and Occupational Therapy, McGill University, Montreal, QC, Canada; 2 Center for Interdisciplinary Research in Rehabilitation, Montreal, QC, Canada; 3 Department of Philosophy, University of Montreal, Montreal, QC, Canada; 4 Faculty of Medicine and Health Sciences, Université Notre Dame d'Haiti, Port-au-Prince, Haiti

**Keywords:** disability, earthquake, Haiti, disaster, vulnerability

## Abstract

**Background:**

Following disasters, persons with disabilities (PWD) are especially vulnerable to harm, yet they have commonly been excluded from disaster planning, and their needs have been poorly addressed during disaster relief. Following the 2010 Haiti earthquake, thousands of individuals experienced acute injuries. Many more individuals with preexisting disabilities experienced heightened vulnerability related to considerations including safety, access to services, and meeting basic needs.

**Objective:**

The objective of this research was to better understand the perceptions of responders and decision-makers regarding disability and efforts to address the needs of PWD following the 2010 earthquake.

**Design:**

We conducted a qualitative study using interpretive description methodology and semistructured interviews with 14 Haitian and 10 international participants who were involved in the earthquake response.

**Results:**

Participants identified PWD as being among the most vulnerable individuals following the earthquake. Though some forms of disability received considerable attention in aid efforts, the needs of other PWD did not. Several factors were identified as challenges for efforts to address the needs of PWD including lack of coordination and information sharing, the involvement of multiple aid sectors, perceptions that this should be the responsibility of specialized organizations, and the need to prioritize limited resources. Participants also reported shifts in local social views related to disability following the earthquake.

**Conclusions:**

Addressing the needs of PWD following a disaster is a crucial population health challenge and raises questions related to equity and responsibility for non-governmental organizations, governments, and local communities.

During disasters and in their aftermath, persons with disabilities (PWD) experience heightened vulnerability to being harmed or wronged (1–4). The ability of PWD to move about or attend to their basic needs may be impeded due to the loss of assistive devices, damage to built and natural environments, separation from caregivers, or being left behind during evacuation. PWD are less likely to be admitted to shelters, receive needed services, or be included in registries of those affected by a disaster. PWD are also more likely to experience abuse, violence, or exploitation. Vulnerability of PWD may be further compounded due to poverty or as a result of combination with other characteristics, for example, vulnerability will be amplified for PWD who are members of a minority group that experiences discrimination (2).

In the past two decades, increased attention has been paid to the ways that disasters impact PWD and to the development of strategies to respond to the needs of PWD in disaster preparedness and response (1, 4, 5). Several initiatives have been undertaken to develop standards of best practices and promote approaches to disaster response that better account for the needs and experiences of PWD (6, 7). The UN Convention on the Rights of Persons with Disabilities also includes the expectation that nations take ‘all necessary measures to ensure the protection and safety of persons with disabilities' in situations of disaster or war (8). Despite these efforts, disaster preparedness, relief, and reconstruction often fall far short of an inclusive approach that is responsive to the diverse capabilities and needs of PWD (3).

Even prior to the 2010 earthquake, PWD in Haiti experienced a range of significant challenges (9). As in other settings, PWD in Haiti are more likely than their fellow citizens to live in extreme poverty (10). Physical and built environments in Haiti also present significant barriers for mobility and inclusion (11, 12); especially for individuals using wheelchairs (13). Many PWD have also experienced discrimination and marginalization (9). Due to these and other factors, PWD in Haiti have less access to education, employment, and health services compared to others in their communities (9). In recent decades, international non-governmental organizations (NGOs) have provided much of the rehabilitation services available for PWD (13–15). Access is often difficult. Bigelow and colleagues identified common challenges accessing prosthetic limbs and follow-up care for amputees, in large part due to a lack of rehabilitation professionals and programs, as well as financial costs that posed barriers for many PWD (12).

On January 12, 2010, a massive earthquake centered near Port-au-Prince resulted in considerable loss of life and widespread destruction. More than 222,000 deaths and 300,000 injuries have been attributed to the earthquake (16). Property and infrastructure were also severely damaged. Many clinics, hospitals, and government ministries were destroyed, as well as thousands of homes and other buildings. About 1.5 million people were internally displaced (17) and as of March 2015 more than 64,000 individuals were still living in camps (17, 18). Immediately following the earthquake, those involved in efforts to help the trapped and injured were predominantly Haitians coming to the assistance of family members and neighbors (19). Many international agencies were already located in Haiti, and also took part in immediate and spontaneous relief efforts. In the subsequent days and weeks, hundreds more military, intergovernmental organizations, and NGOs arrived in Haiti with the goal of contributing to the massive relief, and later reconstruction efforts that were underway (20).

The scale of the destruction wrought by the earthquake was immense, and occurred in a location where infrastructure was already weak, and levels of poverty were high (21). Devastation from the earthquake was particularly widespread due to lack of social services, acute urbanization, and fragile infrastructure; realities that can be traced back to political and historical sources (22). In the face of the destruction, the scale of relief efforts was also enormous, and was carried out by organizations and individuals with widely varying degrees of experience and capacity. Initial relief efforts lacked coordination among NGOs and with the Haitian government, and coordination remained challenging throughout the earthquake response (20). These features contributed to making the provision of coherent and effective aid to all those who needed it difficult to achieve (23).

The 2010 earthquake was both a source of new disability due to injury, as well as a situation that greatly heightened the vulnerability of those with preexisting disabilities (24). Following the earthquake, crush injuries resulted in many amputations being carried out. The frequency of amputations, and discussion of the conditions in which they were performed, became a focus of media attention and a source of considerable discussion within and beyond Haiti (24). Many spinal cord and traumatic brain injuries also occurred. These new injuries resulted in a much higher number of PWD requiring services and support. PWD have continued to experience important challenges in the years following the earthquake, including gaining employment, finding suitable shelter or housing, and accessing health and rehabilitation services (9, 25).

Studies have been conducted that measured rehabilitation services or outcomes following the earthquake (11, 25, 26); also, commentaries were published drawing attention to the importance of coordinated and effective approaches to meet the needs of PWD (4, 15, 27). Knowledge remains limited, however, about how needs of PWD were perceived by those involved in the disaster response. To address this knowledge gap, we analyzed interviews conducted with Haitian and international responders and decision-makers who were involved in the 2010 earthquake response to investigate their perceptions of disability and how the needs of PWD were addressed in relief and reconstruction efforts.

## Methods

We conducted a qualitative study based on interpretive description methodology to examine perceptions of vulnerability in relation to relief and reconstruction efforts following the 2010 Haiti earthquake. Interpretive description is a methodological framework grounded in a constructivist orientation to inquiry (28). Interpretive description methods guide the development of a coherent conceptual account of patterns and commonalities of a particular phenomenon, while accounting for individual variation (29). This framework was developed for use in applied health disciplines and has a particular focus on developing knowledge that can address complex experiential questions related to health care policy and practice. Although we considered diverse sources and experiences of vulnerability in our broader research project, in this paper we present findings related to the perspectives of Haitian and international responders and decision-makers regarding disability and efforts to address the needs of PWD following the Haiti earthquake.

### Participants

Potential participants were identified through investigator contacts, email invitations sent to NGOs, networking through *l'Unité de Recherche et Action Médico Légale* (in Port-au-Prince, Haiti), and a snowball sampling technique where those who participated in the study were asked to suggest others who might be eligible and interested in taking part. To access a breadth of perspectives, we sought to recruit a diverse group of participants across the following dimensions: role within the disaster response, nationality (Haitians and non-Haitians), organizational or institutional affiliation, and sex.

We interviewed 24 individuals who had experience coordinating or implementing elements of the national and international response to the 2010 earthquake. Participants included six former Haitian government officials or decision-makers, five Haitian health professionals who worked with international or national NGOs and civil society organizations (one of whom worked with an organization primarily focused on the needs of PWD), three Haitian health professionals who did not work with an NGO, and 10 individuals from other countries who worked with NGOs involved in the earthquake response (two of whom worked with an organization primarily focused on the needs of PWD). In total, 11 women and 13 men were interviewed. We ended recruitment after interviewing 24 participants because this sample size was consistent with an exploratory interpretive description study (28). We judged the analytic structure to be well developed and that further recruitment would not significantly alter it.

### Data collection and analysis

Each participant took part in an in-depth interview that ranged in duration from 37 to 75 min (mean of 60 min). Two-thirds of the interviews were conducted in person and the remainder by phone or Skype. Interviews were conducted in English or French per the preference of the participant and followed a semistructured interview guide. The interviews were audio recorded and transcribed. A member of the research team reviewed the transcripts to ensure their accuracy. A synopsis of each transcript was then written that highlighted key elements of each interview, and emerging insights and ideas. NVivo software was used to organize the coding process. A member of the research team initially coded three transcripts. Provisional codes were then reviewed with other members of the team, and a coding structure was developed collaboratively. All transcripts were then analyzed based on this coding scheme, which was further revised in an iterative process. Constant comparative techniques were used to compare data within a single transcript and across transcripts (30). Throughout the analysis we sought to maintain attention to the role and position of participants (Haitian government official, international NGO worker, etc.).

For the analysis of findings related to disability, we developed categories and themes related to perceptions of disability and how the needs of PWD were addressed in the earthquake response. Development of categories was undertaken by asking the following questions: What patterns and linkages emerge from the data related to disability? What explains these patterns? To test the provisional categories, we asked the following question: What does not fit or is left aside by this analytic structure? Themes were then developed through an inductive process by asking the question: What concepts/ideas capture the core aspects of the phenomenon of interest? All analytic units (codes, categories, themes) were developed in English across both English and French transcripts to develop a consistent analytic structure.

All participants read and signed an informed consent form. The study was approved by the National Bioethics Committee of Haiti, and research ethics committees at McGill University and the University of Montreal.

Selected verbatim quotations are included in the results section to illustrate aspects of the analysis. Quotations included in the results section that were originally in French were translated into English by a professional translator.

## Results

Study participants discussed diverse sources of vulnerability following the earthquake including heightened vulnerability for children, women, older people, individuals who were very poor or who lacked social supports, and individuals who had a chronic illness or were living with HIV/AIDS. Among these more general discussions of vulnerability, participants consistently identified PWD as a group who experienced high levels of vulnerability in the days and months following the earthquake. This view of the heightened vulnerability of PWD is reflected by two Haitian participants who emphasized that PWD were among the most vulnerable individuals following the earthquake. The first stated that PWD were ‘… people … who are the most vulnerable of the vulnerable'. The second, who worked with an international NGO specializing in the area of disability, expressed, ‘I can honestly say I saw people who were really very, very, very, very, very vulnerable'.

Several participants also discussed variations in the degree and nature of vulnerability experienced by PWD. The Haitian participant who worked with a disability-focused NGO described how PWD are a diverse group who experienced different levels of vulnerability and had different needs. This diversity went beyond the more descriptive categories established by her organization which included ‘… general criteria, but on top of that, people who were injured, people who were bedridden, injured people who hadn't received any care, bedridden people who had no one to help them move about'. The narratives offered by the other participants who worked directly with PWD also reflected how particular impairments could lead to different degrees or forms of vulnerability, as well as vulnerabilities being shaped by other characteristics including social network, sex, and level of poverty. Thus, while PWD were often referred to as a single, unified group, several participants introduced greater nuance when discussing the capacities and needs of individuals based on their impairments and particular circumstances.

Through a process of inductive analysis, we identified three core themes related to disability and PWD: 1) Attention given to disability: disability on and off the map, 2) Challenges to addressing the needs of PWD in relief efforts, and 3) Reconstruction, social realities, and changing perceptions.

### Attention given to disability: disability on and off the map

Participants reported that following the earthquake, disability received increased attention from multiple sources, including international media, donors, NGOs, and within Haitian society. An experienced humanitarian worker described that this situation was distinctive compared to other situations of crisis: ‘It was the very first time … I think it was everyone's agenda to attend to people who were injured … So acute disability was really on the map'. This participant suggested that the level of attention given to PWD following the Haiti earthquake contrasted with other major disasters where disability was little discussed in the media or by major aid agencies, remaining a niche concern for specialized organizations. Increased attention to disability was attributed primarily to the frequency, but also the prominence, of acute injuries leading to amputations and spinal cord injuries resulting from the earthquake. Several participants further suggested that increased attention to PWD by aid agencies was in part the result of the attention the media and, subsequently, aid donors paid to disability. Greater attention to PWD within Haitian society was also reported. This phenomenon is reflected by a Haitian government official who expressed that after the earthquake there was a sense that, ‘So, there was this new, enormous category of people with disabilities'. The participant linked new injuries from the earthquake to a greater awareness of disability and PWD within Haitian society.

Yet, the international humanitarian worker who described acute disability as being on the map following the earthquake went on to suggest that the attention that was given to disability was in fact quite narrow, being primarily focused on amputees and individuals who had sustained spinal cord injuries. Although acknowledging the importance of these conditions and the need to assist individuals who had been injured, a second international humanitarian worker described how this focus sometimes led to a mismatch between needs and services. This participant worked directly with PWD who would have benefited from bracing or assistive devices. She reported that many of these supports could have been fabricated in one of the specialized orthotics/prosthetics workshops that were established after the earthquake but these individuals' needs ‘were not aligned with the priorities of the donors supporting these projects, which were directly aligned with the international attention that was given to amputees. It was not given to people with other orthopedics problems'. As a result ‘… there were people with clearer needs and vulnerabilities whose needs were not being addressed because they didn't fit into the right category or box'. This participant expressed frustration with the narrow scope of needs that were prioritized and which seemed to her more driven by donor priorities than by population needs, resulting in a ‘… a severe example of this … high-publicity, short-term um, kind of fly-by-night humanitarian response'. Thus, although acute disability was now ‘on the map' and an identified priority within the response, only some forms of disability were considered a priority, leading to a situation where individuals with certain conditions had access to specialized services and support, while others did not.

### Challenges to addressing the needs of PWD in relief efforts

Although acknowledging that PWD faced distinctive obstacles to access services and have their basic needs met, participants reported divergent views and practices of how PWD were included in relief and early reconstruction efforts, and multiple challenges to addressing the needs of PWD. Addressing the needs of PWD was associated with multiple sectors of the aid response, and doing so effectively was seen as requiring a coherent, integrated strategy across these domains that was difficult to achieve. Participants identified a range of areas that required attention in relation to the needs of PWD, including health care and rehabilitation services, shelter (both temporary shelter in the camps and new constructions that would be accessible for people with mobility restrictions), and the provision of essential supplies (e.g. organizing distribution sites for food and water to be accessible for PWD).

In considering how to address the needs of PWD, two perspectives were described: seeing disability as a niche concern to be addressed by organizations specialized in working with PWD, or as something that all organizations should take into account in every program. For example, although many participants working with international aid organizations described that their NGO prioritized vulnerable groups, and included PWD within these priority groups, they rarely offered examples of how this prioritization was reflected in the organization's field projects even when invited to do so. Participants who worked for NGOs whose mandate was focused on PWD also reported they sometimes experienced limited receptivity when advocating that other organizations adapt their programs and methods to be more inclusive. A participant thus reported that an organization might have replied in the following way to the suggestion that they adopt strategies to be more inclusive in their feeding programs: ‘… we'll feed the way we know how to feed and, because you work with disability, we'll leave that to you'. Although many individuals and organizations were more open to the encouragement to find ways to be more inclusive, she expressed that she and her colleagues ‘had to be very vocal' in their advocacy efforts, yet still had limited success in influencing the practices of some agencies. This participant, and another who had extensive experience working with PWD, expressed the view that disability ought to be further integrated within the response of all organizations.

Narratives recounted by participants also reflect how the varied needs among PWD required a differentiated approach – and how tailored efforts to address the needs of PWD could be a source of equity concerns in a context where vulnerability is widespread. For example, an international participant described how the use of a siren to warn of a potential hurricane risked excluding individuals who were deaf. Challenges were also encountered in some initiatives that specifically targeted PWD. The same participant described how her organization planned to preevacuate residents with mobility limitations from a camp that was at risk for flooding. In a context of rising frustration and distrust, other residents of the camp refused the pre-evacuation. She described how: ‘… the other people in the camp came forward saying, “nobody is leaving this camp unless you evacuate all of us,” and there was like, 50 000 people …' However, the participant suggested that this situation also raised a broader normative question: ‘So it was how do we approach the situation, and is it ethical to take the most vulnerable when everybody is in the same vulnerable situation?' This experience reflected a core challenge related to providing different services or resources to PWD in a situation where many felt desperate for help, and were not confident that assistance would be forthcoming.

In considering the needs of PWD, participants also discussed limits to what types of tailored programs would be justified in a situation of scarce resources. Several participants referred to the added costs of building housing that would be accessible to wheelchair users or others with mobility limitations. Questions were raised about what types and extent of adaptation would be justified if it meant that fewer shelters could be constructed due to added costs or lengthier construction time. These questions were not just raised in retrospect but appeared to be a source of active discussion during the relief phase; a participant reported that the reality that needs vastly exceeded resources, and resources needed to be stewarded carefully, resulted in ‘the overarching ethical dilemma that we probably faced every day'.

Two interlinked concerns further limited the effectiveness and extent of services for PWD: lack of coordination and difficulties with information sharing. Participants reported challenges in coordinating services for PWD. It was noted that this issue was not specific to disability; coordination was a widespread challenge in all facets of the earthquake response. Coordination of services for PWD was initially hampered by the lack of a mechanism to share information. A Haitian participant reported that

… in the beginning, … we didn't have the same information, but after a while we were able to … collect all the information to some extent, and also, people were moving from place to place. There were different ways, different organizations providing services. And the services weren't being documented.

An international participant further noted that such challenges were heightened due to the presence of many smaller organizations arriving for short duration trips to provide rehabilitation services. Given the barriers to sharing information and coordinating between agencies, a participant reported that to locate and assist PWD in the early days after the earthquake she and her colleagues often relied on neighbors in the camps, or even strangers, who would bring information to them about the needs of PWD. The Haitian participant who reported barriers to information sharing in early relief phase efforts indicated however that coordination improved over time, particularly with the development of rehabilitation sector cluster meetings, which brought together organizations to share information and coordinate their programs.

### Reconstruction, social realities, and changing perceptions

While discussing diverse sources of vulnerability, including disability, participants reflected on the importance of considering longer-term impacts of projects, and the need to take into account social realities. However, several participants described that projects initiated in the relief phase often only considered the short term, and even many reconstruction projects did not address broader social considerations related to vulnerability. An international participant described how her hopes that an organization involved in providing prosthetic limbs would consider the broader social context were largely disappointed: ‘I was hoping that they would integrate the care of their beneficiaries with the social reality … and I, I found that to be a losing battle'. Providing adaptive aids or rehabilitation services was thus seen by several participants as important but far from sufficient to meet the needs of PWD. For example, participants acknowledged an array of considerations including the importance of changing legal provisions, promoting employment opportunities, and creating opportunities for access to education, as means to achieve greater inclusion of PWD in their communities. A Haitian participant emphasized how broader questions of inclusion – and collective responsibility – were raised for Haitian society after the earthquake: ‘We had never before had so many … had so many disabled persons as … we did then! And, in fact, we were faced with problems, such as, how do we reintegrate these people into society?' From this perspective, working toward social inclusion was seen as a crucial but daunting objective.

As this participant described, after the earthquake Haitian society was faced with important questions in relation to PWD. This situation appears to have had an influence on social perceptions of disability. Several participants, both from Haiti and other nations, suggested that there have been alterations to how disability is viewed in Haiti. These participants reported that having a disability was associated for many people with feelings of guilt or embarrassment, seen as something to be kept hidden, and referred to in a pejorative manner. Participants reported, however, that social perceptions in regards to disability had shifted somewhat since the earthquake. A Haitian participant described that:

You see, it was – before, there was … an attitude of rejection. Now, it's accepted … it's not always easy, it attracts attention, but there are fewer negative comments associated with it … And also, people go out, we see a lot more people with chronic conditions, now.

Another reported that PWD are less hidden within society and that ‘… people with disabilities are less … that is, it's not such a disgraceful thing anymore'. He went on to say that ‘… these days, it's not as shocking' to see someone with a disability because it is more common since the earthquake.' Two participants linked these changes to increased visibility of individuals with disabilities, including individuals who were injured during the earthquake and who resumed positions of influence within the community, particularly in government. A Haitian participant described how ‘… there are lots of people who went back to their jobs … especially in the civil service'. An international participant also expressed that:

… with the earthquake, what we did see is that perceptions changed because everybody knew somebody who um, who was either you know, um, acutely injured after the earthquake, or will have long term sustained injuries as a result of the earthquake. So the acceptance of persons with disabilities definitely increased after the disaster.

Thus, the earthquake was seen as a trigger for social changes of how PWD were perceived.

## Discussion

In a wide range of fields including health policy, disaster studies, and bioethics, the notion of vulnerability is used to characterize individuals or groups of individuals with increased likelihood to experience harm or be wronged (31–33). This understanding is consistent with the views expressed by participants in our study, some of whom defined the concept of vulnerability in relation to identification with a group of people who were all seen as collectively experiencing greater risk of being harmed or excluded (e.g. children, individuals who are poor, women, PWD). In the narratives of the participants, vulnerability was seen as both an inherent human characteristic and as resulting from particular circumstances, institutional structures, and social arrangements. Thus, vulnerability to injury or death due to the devastation caused by an earthquake was seen as a generalized human susceptibility. At the same time, vulnerability was understood to vary considerably between individuals, including among PWD, depending on many other factors such as where they lived; their sex; their state of health; their experience of physical, sensory, or intellectual impairments; their experience of exclusion or isolation; and the strength of their social network. These observations reflect an understanding of vulnerability as dynamic and situational, and shaped by particular circumstances, social structures, and institutional features (3, 34). A tension exists, however, between this more nuanced appreciation of the ways that individuals experience vulnerability and the use of the category of a vulnerable group. Although not unique to disaster response, this tension is reinforced by the use of vulnerable groups as an operational category linked to organizational mandates and prioritization models. However, when groups of people are seen as collectively vulnerable, there is a risk that individual experience and intersections among diverse characteristics will be concealed.

In the aftermath of the Haiti earthquake, amputations and spinal cord injuries received considerable attention and many new rehabilitation and prosthetic programs were launched to address the important needs in these areas (24). There were, however, fewer services available for PWD who were not amputees and few programs to promote the social inclusion of PWD. The emphasis on amputations and other acute injuries reflects, in part, the mandates of some organizations responding to natural disasters to treat only ‘disaster-related' injuries (35). This focus appears to have been reinforced by the strong media focus on amputees. Following a large-scale disaster, individuals with a wide range of impairments, including those that preceded the disaster, will also need rehabilitation services and other forms of support. Tataryn and Blanchet (24) report that 4 months after the Haiti earthquake, more than half of individuals using rehabilitation services set up after the earthquake had a preexisting disability. As emphasized by the participants in our study, it is crucial to pay attention to the needs of individuals with a wide range of preexisting impairments as well as new injuries. In this light, those providing and those funding rehabilitation services following a disaster in a setting where rehabilitation services were limited beforehand should take account of the potentially significant needs of all PWD in the planning, staffing, and equipping of their programs.

Concerns raised by participants regarding a lack of coordination of interventions in the rehabilitation sector, which resulted in limited effectiveness in meeting the needs of PWD, have been discussed elsewhere (36). Tataryn and Blanchet (24) note that there were more than 125 organizations involved in rehabilitation activities postearthquake, and that services were impeded by a lack of efficient coordination between organizations, difficulties with record keeping and data collection, and a mismatch between resources that were available (including inappropriate donations of material) and actual needs. They further describe disability as being addressed with a silo approach by specialized organizations rather than as a more transversal consideration across all agencies. This assessment corresponds with the tension identified by our participants between disability as a niche concern for specialty organizations or a mainstream consideration. The difficulty of coordinating services for PWD was further heightened by the need for an intersectoral approach. This reality reflects the inherent complexity of disaster management at the micro level. To work effectively in such environments, organizations require situational awareness, collaborative approaches, responsive planning, and an appreciation for the dynamic nature of the context (37). Although the Haiti earthquake response incorporated more attention to disability than in most prior disasters (38), there were still significant unmet needs (9, 24). Renewed effort is required to operationalize standards and guidelines that have been developed for addressing disability in disaster response, improve coordination between organizations and across aid sectors, and provide more coherent and effective services for PWD in contexts that are complex and often chaotic (2, 6, 8).

PWD in Haiti have tended to experience marginalization, and encounter barriers to accessing education, work, and needed health care services (9). One of the particularly interesting findings from our research is the set of reflections around changing social perceptions of PWD following the earthquake. Links were suggested between the return to work of PWD injured in the earthquake, particularly individuals working in high visibility jobs such as for the government, and how PWD were seen by the community. It is noteworthy that, in this sense, increased inclusion in the employment sector led to greater social acceptance of PWD, rather than greater acceptance leading to increased inclusion. Although incremental change was described, PWD continue to experience barriers. As Danquah and Brus reported in 2013, after the earthquake, ‘people with disabilities interviewed said that the attitudes of the people around them at home, at school, and at work limited their involvement in activities that were important to them' (9). In a study of amputees receiving rehabilitation services and prosthetic limbs following the Haiti earthquake, Campbell et al. (39) report that although participants felt gratitude for the help they received to relearn how to walk, they also expressed a broader concern about social inclusion and how they would ‘relearn to live' with a disability. This observation is supported by the participants in our study who underlined the importance of social inclusion but were uncertain about how this objective could best be supported.

Peter Redfield has described humanitarian crises – especially acute emergencies such as the Haiti earthquake – as providing a seductive ‘moral clarity' (40). One aspect of this apparent clarity is the way that utilitarian calculation is used to seek to provide the most help to the greatest number. This approach largely focuses on the distribution of resources or triage of services, and individuals may be seen as undifferentiated units of resource distribution with limited attention to their particular capacities. The present research helps to illustrate some of the ways that this apparent moral clarity may mask important ethical concerns for equity, including how vulnerabilities shape people's access to and capacity to make use of the resources and services provided during a crisis (41). This reality is clearly illustrated by the situation of PWD following the Haiti earthquake. Especially as an acute disaster abates, the ethics of catastrophe (42) ought to cede way to other approaches that better account for contextualized vulnerabilities, and the possibility of injustices that may result from treating individuals as generic disaster ‘victims'. The capabilities approach (43) helps draw attention to these concerns. The notion of capabilities relates to an individual's ability to make use of resources for purposes that she or he deems important. This approach is thus individual-centered rather than resource-centered in its focus, which better accounts for differences between individuals (in terms of their goals and objectives, but also their capabilities to access and use resources) and is less passive. Focusing on capabilities also reveals the various determinants that shape a person's ability to utilize resources to advance her or his own ends. A capabilities approach to relief and reconstruction would thus seek to identify obstacles and barriers that prevent individuals, including PWD, from achieving their capabilities in the aftermath of disaster. It also suggests that those providing assistance be vigilant to the possibility of inequities, especially those that might be created or sustained by their own practices and policies.

This paper presents findings related to disability and the needs of PWD following the 2010 earthquake as perceived by Haitian and international responders and decision-makers who were involved in coordinating or providing services. These results arise from a larger project on vulnerability and the Haiti earthquake. As a result of the project's design and primary objectives, this study did not solicit perspectives of those most directly affected by issues of disability and inclusion; that is, PWD and representatives of disabled person's organizations. Additional inquiry into changing social perceptions of disability in Haiti would be a valuable contribution to better understand the phenomenon suggested here. Such research, however, should incorporate the perspectives of PWD themselves, and explore how they experience and understand any alterations in social perception. Additional research to evaluate the effectiveness of different models to address the needs of PWD in relief and reconstruction projects for future disaster events is also warranted. Such research would make an important contribution to expanding the evidence base related to best practices in meeting the diverse needs of PWD during disasters – including research that extends beyond rehabilitation services and considers broader questions of inclusion and sustainability (44).

## Conclusions

This inquiry points to the reality that the effects of so-called ‘natural' disasters are the result of historical, social, and technological features that shape how societies are organized, buildings constructed, and relief efforts are organized and implemented (42). The 2010 Haiti earthquake illustrates the impact of these features and how they shape vulnerabilities, including those experienced by PWD. Although there have been several efforts to promote best practices and develop guidelines to better address the needs of PWD in disasters, there remain significant obstacles to the implementation of disaster preparedness, relief, and reconstruction that is inclusive of PWD and responsive to their needs. Developments in recent years including the UN Convention on the Rights of PWD (8) and the Bonn Declaration (6) in 2007, as well as the World Health Organization guidelines in 2013 (7), are important steps forward, yet more work is needed to ensure that inclusive approaches to disaster response will be more widespread, well-coordinated, and comprehensive in the future. At the same time, critical reflection is needed regarding the ways that the concept of vulnerability and the category of vulnerable groups are used in disaster response initiatives. Concerns include the possibility that the diverse capacities and needs among individuals identified as being part of a vulnerable group may be glossed over, and that the use of narrow categories may lead to the exclusion of others who also experience elevated needs. Broader discussion within the humanitarian community, efforts to advocate for more attention to disability, and targeted research on disaster and disability can all serve to advance the goal that national and international responses to future disasters will be characterized by increased attention to the needs of all PWD.
